# Use of antibiotics in newborns with infections at rural referral hospital in Uganda: compliance with empirical guidelines

**DOI:** 10.3389/fped.2026.1857839

**Published:** 2026-09-09

**Authors:** Ellen Andersson, Santorino Data, Lea Kreyenbaum, Khakusuma Shanita Shamusa, Ashish KC

**Affiliations:** 1 School of Public Health and Community Medicine, Sahlgrenska Academy, University of Gothenburg, Gothenburg, Sweden; 2 Department of Paediatrics and Child Health, Mbarara University of Science and Technology, Mbarara, Uganda

**Keywords:** adherence to protocol, antibiotics, neonatal sepsis, possible severe bacterial infection, rural Uganda

## Abstract

**Introduction:**

Despite global progress, neonatal infections remain a major cause of mortality in low- and middle-income countries. In Uganda, high neonatal mortality and rising antimicrobial resistance underscore persistent challenges in prescribing appropriate treatment. WHO developed a Global Action Plan to promote safe and effective antibiotic use, which forms the basis of Uganda's National Action Plan. Limited evidence exists on how national guidelines are followed, particularly regarding first-line therapy and dosing. The aim is to assess adherence to national guidelines for inpatient use of antibiotics in neonates with suspected infections at Mbarara Regional Referral Hospital (MMRH), and factors affecting the adherence.

**Methods:**

This cross-sectional study included 300 neonates admitted to the Neonatal Intensive Care Unit at MRRH Uganda, between October and November 2024. Clinical records were analysed to summarize demographics, danger signs, diagnoses, and antibiotic use by WHO Possible Severe Bacterial Infection (PSBI) criteria. Logistic regression was done to assess the predictors for compliance for antibiotics.

**Results:**

Of the total population (*n* = 300), 125 (41.7%) had ≥2 DSs, 124 (41.3%) had 1 danger sign, and 51 (17%) had none. In total, 96.7% received antibiotics, and 16.6% of these had no danger sign. Among those who received antibiotics and had ≥1 danger sign, 58.3% received recommended type and dose. Among those with ≥ 2 danger sign, 98.4% received any antibiotic, 80% received the first-line combination, and 60.8% the correct dose. Higher number of danger sign (OR = 7.96, 95% CI 4.76-13.30, p = <0.001), and the presence of hypoxic ischemic encephalopathy (OR = 4.74, 95% CI 1.13–19.89, *p* = 0.033), and birth asphyxia (OR = 6.23, 95% CI 1.30–29.83, *p* = 0.022) were associated with higher adherence. No blood cultures were performed.

**Discussion:**

Low compliance with guidelines in cases of PSBI and frequent use of antibiotics in neonates with no or only one danger sign was observed. However, only one danger sign is an indication for antibiotics in Uganda's guidelines, indicating that national guidelines may lead to broader antibiotic use than WHO recommendations.

## Introduction

Neonatal infections remain a major global public health concern and are a leading cause of neonatal morbidity and mortality. In 2019, an estimated 5.3 million children under five years of age died worldwide, of whom 46% were neonates, and 49.2% of neonatal deaths were attributable to infections (1). Low- and middle-income countries (LMICs) account for approximately 99% of these deaths, over 80% of which are considered preventable through timely and appropriate care (2). Alongside this burden, antimicrobial resistance (AMR) has emerged as a major threat to effective infection management. In 2021, bacterial AMR was associated with an estimated 4.7 million deaths globally and was directly responsible for 1.1 million deaths, with projections indicating a continued increase in AMR-attributable mortality in the coming decades (3).

Global efforts to reduce child and neonatal mortality have demonstrated that coordinated strategies can yield substantial improvements. Building on the progress of the Millennium Development Goals (MDGs), the 2030 Agenda for Sustainable Development emphasizes integrated approaches to health, equity, and well-being (4). SDG 3 specifically targets the reduction of preventable neonatal and under-five mortality, with neonatal mortality rate serving as a key indicator (4). Sub-Saharan Africa continues to carry the highest burden of neonatal mortality, with slower rates of decline compared to other regions (11). This region also experiences high rates of prematurity and small-for-gestational-age births, both of which increase vulnerability to neonatal infections and death. Although Uganda has made progress toward SDG 3, achieving approximately 50%–55% of the required progress by 2025, neonatal mortality remains a major challenge, underscoring the need for targeted interventions (5). In Uganda, neonatal mortality has declined from 98 deaths per 1,000 live births in 1988–1989 to 18 per 1,000 live births in 2022 (6). Despite these improvements, 75% of neonatal deaths occur within the first week of life. Common causes of neonatal admission at Mbarara Regional Referral Hospital (MRRH) include prematurity, low birth weight, sepsis, hypothermia, and birth asphyxia, with hypothermia and neonatal respiratory distress syndrome being major contributors to mortality (7). Studies from other regions in Uganda report high proportions of neonatal admissions due to sepsis, highlighting infections as a persistent driver of neonatal intensive care utilization (8).

Neonates are particularly susceptible to infections due to immunological immaturity, incomplete physical barriers, and reliance on maternal antibodies for early immune protection (9). In LMICs, this vulnerability is compounded by higher rates of malnutrition and undernutrition, which further impair immune function (10). Early detection and prompt treatment of infections are therefore critical. In resource-limited settings, the World Health Organization (WHO) recommends the use of simplified clinical criteria for possible severe bacterial infection (PSBI), based on observable danger signs (DS), to guide empirical antibiotic treatment (2). While diagnostic tools such as blood cultures and biomarkers improve diagnostic accuracy, their use is often limited by cost, infrastructure, and availability of trained personnel (11).

Preventive strategies, including improved hygiene, infection control practices, maternal screening, and vaccination, play an important role in reducing neonatal infections and antibiotic use (12, 13). Nevertheless, antibiotics remain central to neonatal infection management. Although lifesaving, antibiotic overuse and misuse are associated with adverse outcomes, including disruption of the neonatal microbiota and increased risk of necrotizing enterocolitis, particularly among very low birth weight infants (14). Diagnostic uncertainty and limited laboratory capacity contribute to widespread empirical antibiotic use in LMICs, raising concerns about unnecessary exposure and resistance development (15).

WHO recommends empirical treatment of neonatal sepsis with ampicillin and gentamicin, supported by blood culture testing prior to antibiotic initiation where feasible (16). Uganda's national clinical guidelines largely align with WHO PSBI criteria but permit antibiotic initiation based on a single DS and recommend alternative regimens under specific clinical conditions (17). While these adaptations aim to reduce missed infections, they may also contribute to increased antibiotic use.

AMR represents a growing challenge in Uganda. The country has a high burden of AMR-related mortality, with thousands of deaths directly attributable to resistant infections each year (18). Studies from southwestern Uganda have reported alarmingly high prevalence of extended-spectrum beta-lactamase-producing organisms, limiting treatment options (19). Although Uganda adopted a National Action Plan (NAP) on AMR in 2018, implementation remains constrained by limited resources, awareness, and surveillance capacity (20, 21). Antibiotic consumption in Uganda is high compared to global averages, and adherence to national treatment guidelines remains suboptimal in hospital settings (22, 23).

Despite the existence of national and international guidelines, there is limited evidence on how consistently these recommendations are implemented in neonatal care in low-resource settings. Notably, adherence to first-line antibiotic choice and correct dosing among neonates with suspected infection remains underexplored. No previous study has evaluated antibiotic use and guideline adherence for neonatal infections at MRRH, representing a critical gap in knowledge. MRRH was selected as the study site due to its role as a high-volume regional referral hospital serving south-western Uganda and neighboring countries, making it a strategically important setting in which to assess real-world adherence to antibiotic guidelines. Uganda's recommended first-line empirical regimen for neonatal infection comprises ampicillin (50 mg/kg/dose, 12-hourly in the first 7 days) and gentamicin (5 mg/kg/dose, once daily in first 7 days), initiated upon identification of at least one WHO-defined danger sign per national guidelines (17).

Therefore, this study aimed to evaluate adherence to recommended antibiotic therapy including type and dosing among neonates admitted with suspected or confirmed infection at MRRH. In addition, it sought to describe patterns of antibiotic prescription, quantify treatment gaps, and identify clinical and demographic factors, including DS and neonatal diagnoses, associated with guideline-adherent therapy. The findings are intended to inform improvements in neonatal infection management and support antimicrobial stewardship efforts.

## Materials and methods

### Study design

This study was a hospital-based cross-sectional study among newborn children (0–28 days) hospitalized at the Neonatal Intensive Care Unit (NICU) at MRRH, Uganda, between October 2024 and November 2024. The study used clinical records that routinely collected the presence of neonatal DS, antibiotic prescriptions, diagnoses at admission, and basic demographics.

A retrospective design was chosen to provide a descriptive overview of clinical management during a defined time period without interfering with clinical practice. All neonates admitted during the study period were screened for eligibility, and a census approach was applied to ensure that findings reflect real-world care in this setting.

### Setting

MRRH is a government owned hospital in Mbarara City in the South-Western part of Uganda. The hospital serves a large catchment area, and covers the Ankole sub-region, neighboring districts and neighboring countries including Rwanda, Democratic Republic of Congo, Burundi and Tanzania. The hospital covers a wide range of departments including Emergency, Mother Child Health (MCH), Maternity, Pediatric ward and surgery, Pediatrics Oncology, Radiology and Intensive Care Units (ICU), and many more.

### Study population

The study population consisted of 300 neonates admitted to MRRH between October 2024 and November 2024. Inclusion criteria were neonates with clinical signs of newborn infections according to WHO's definition of PSBI. This definition requires at least 2 out of 6 clinical signs, including fast breathing (respiratory rate ≥60), severe chest indrawing, fever (≥37.5℃) or hypothermia (<35.5℃), no movement at all or only at stimulation, feeding poorly or not at all, and history of convulsions.

### Data collection

Data were collected from the NICU PRISM (Protecting Infants Remotely by SMS) database where the clinical assessments for newborn infections were routinely captured prospectively. Before data extraction, all patient records were anonymized using a master register in which unique study identifiers replaced personal information to ensure confidentiality. Relevant variables were then retrieved using a structured data extraction tool. The extracting-tool was designed to systematically capture information on demographic characteristics, clinical presentation, DS, antibiotic treatment, and outcomes. Data extraction was carried out manually and cross-checked by another person.

### Data analysis

All data were systematically cleaned and coded, including checks for missing and inconsistent values. Missing data were retained as such, and no imputation procedures were applied. All participants were preserved in the dataset; exclusions were performed only within specific analyses where variables of interest contained missing values. Categorical variables were recoded, and derived variables were generated where appropriate. In addition, all variables were consistently labelled to enhance clarity. Data validation procedures were implemented to ensure accuracy and consistency prior to statistical analysis. Data validation included cross-checking extracted antibiotic entries against original records by a second reviewer (LK), and range checks on dosing values.

The primary outcome was full adherence, defined as prescription of both ampicillin AND gentamicin at correct weight-based doses per Uganda national guidelines (17). Dosing correctness was assessed by comparing prescribed doses against weight-based reference values per the Uganda Clinical Guidelines 2023: ampicillin 50 mg/kg/dose and gentamicin 5 mg/kg/dose (once daily), with actual body weight at admission used as the reference. Neonates for whom body weight or prescribed dose was not documented were excluded from dosing analyses, and the number of participants available for each specific analysis is provided in the results. Descriptive analyses were conducted to summarize neonatal characteristics, clinical signs, and antibiotic prescription patterns, stratified by indication status according to the World Health Organization (WHO) criteria for PSBI. Logistic regression was applied to identify predictors among participants who met WHO PSBI criteria and demonstrated full compliance. Logistic regression was conducted in two steps: univariate analyses were first performed for each candidate variable, followed by a multivariable model including all variables. Results are expressed as odds ratios (OR) with 95% confidence intervals (CI). Results are presented using descriptive tables and illustrative figures. All analyses were performed using Stata/SE version 19.5.

### Ethics

The data was collected from a patient register and analyzed on group level and not by individuals. Each patient record was anonymized and designated with a study-ID only able to track through a master register. Prior to data-collection, consent was obtained from the hospital management for the use of medical records. The highest ethical standards were applied in the process of collecting data from these files. The research was approved by the Research Ethics Committee of MUST, approval number MUST-2025-507, and registered with the Uganda National Council of Science and Technology (UNCST). To guide data ownership, storage and publication rights, a data sharing agreement was obtained between MUST and University of Gothenburg. The study was conducted in accordance with the ethical principles of the Declaration of Helsinki.

## Results

A total of 300 neonates admitted to the NICU were included, of whom 17% had no DS, while 41.3% and 41.7% presented with one and ≥2 DS, respectively. More than half of admissions occurred within the first day of life (55%), with a similar age distribution across groups. Overall, 55.7% of neonates were male. The majority had a birth weight of 2,500–3,999 g (60.3%), while 12.7% weighed <1,500 g (see Table 1).

**Table 1:** Characteristics of neonates admitted to the NICU.

Characteristics	Total population	Population with no DS^a^	Population with 1 DS^a^	Population with ≥2 DS^a^
n (%)	300 (100)	51 (17%)	124 (41.3%)	125 (41.7%)
Age, n(%)				
1 day	165 (55)	28 (55)	67 (54)	70 (56)
2–6 days	118 (39.3)	17 (33.3)	54 (43.5)	47 (37.6)
7–13 days	8 (2.7)	3 (5.9)	1 (0.8)	4 (3.2)
14–28 days	9 (3)	3 (5.9)	2 (1,6)	4 (3.2)
Sex, n(%)				
Male	167 (55.7)	31 (60.8)	62 (50)	74 (59.2)
Female	133 (44.3)	20 (39.2)	62 (50)	51 (40.8)
Weight, n(%)				
< 1,500g	38 (12.7)	1 (2)	14 (11.3)	23 (18.4)
1,500–2,499g	60 (20)	12 (23.5)	23 (18.5)	25 (20)
2,500–3,999g	181 (60.3)	28 (54.9)	77 (62.1)	76 (60.8)
>4,000g	21 (7)	10 (19.6)	10 (8.1)	1 (0,8)
Place of birth, n(%)				
Hospital	284 (94.7)	46 (90.2)	122 (98.4)	116 (92.8)
Home	8 (2.7)	3 (5.9)	1 (0.8)	4 (3.2)
Clinic	8 (2.7)	2 (3.9)	1 (0.8)	5 (4)
Danger signs, n(%)				
Not feeding well	167 (55.7)	-	61 (49.2)	106 (84.8)
History of convulsions	16 (5.3)	-	2 (1.6)	14 (11.2)
No movement/only at stimulation	5 (1.7)	-	1 (0.8)	4 (3.2)
Fast breathing (≥60)	93 (31)	-	17 (13.7)	76 (60.8)
Severe chest indrawing	56 (18.7)	-	4 (3.2)	52 (41.6)
Fever (≥37.5℃)	75 (25)	-	36 (29)	39 (31.2)
Hypothermia (<35.5)	18 (6)	-	3 (1)	15 (12)
Mean amount of DS, n	1.43	-	1	2.45
Diagnoses at admission, n(%)				
Sepsis	65 (21.7)	6 (11.8)	34 (27.4)	25 (20)
Prematurity	64 (21.3)	10 (19.6)	25 (20.2)	29 (23.2)
NRDS^b^	40 (13.3)	4 (7.8)	9 (7.3)	27 (21.6)
Jaundice	31 (10.3)	5 (9.8)	17 (13.7)	9 (7.2)
HIE^c^	17 (5.7)	2 (3.9)	4 (3.2)	11 (8.8)
Macrosomia	17 (5.7)	9 (17.6)	7 (5.6)	1 (0.8)
Birth asphyxia	13 (4.3)	3 (5.9)	2 (1.6)	8 (6.4)
LBW^d^	10 (3.3)	1 (2)	4 (3.2)	5 (4)
Others	35 (11.7)	6 (11.8)	17 (13.7)	12 (9.6)
Not recorded	75 (25)	10 (19.6)	33 (26.6)	32 25.6)
Congenital anomalies, n(%)				
Yes	19 (6.3)	2 (3.9)	4 (3.2)	13 (10.4)
No	279 (93)	48 (94.1)	120 (96.8)	111 (88.9)
Not recorded	2 (0.7)	1 (2)	0 (0)	1 (0.8)
Receiving antibiotics, n(%)				
Yes	290 (96.7)	48 (94.1)	119 (96)	123 (98.4)
No	10 (3.3)	3 (5.9)	5 (4)	2 (1.6)
Blood cultured, n(%)				
Yes	0 (0)	0 (0)	0 (0)	0 (0)
No	300 (100)	51 (100)	124 (100)	125 (100)
Outcome, n(%)				
Alive	258 (86)	43 (84.3)	107 (86.3)	108 (86.4)
Died	12 (4)	1 (2)	1 (0.8)	10 (8)
Absconded	1 (0.3)	1 (2)	0 (0)	0 (0)
Referred	1 (0.3)	0 (0)	0 (0)	1 (0.8)
Not recorded	28 (9.3)	6 (11.8)	16 (12.9)	6 (4.8)

^a^ DS, danger signs; ^b^NRDS, neonatal respiratory distress syndrome; ^c^HIE, hypoxic ischemic encephalopathy; ^d^LBW, low birthweight.

* Neonates could have more than one diagnosis at admission, so numbers in the diagnoses section do not sum to the group totals.

Most neonates were born in hospitals (94.7%), with very few home or clinic births. The prevalence and severity of clinical DS increased markedly with the number of DS: not feeding well, fast breathing, and severe chest indrawing were substantially more common among neonates with ≥2 DS. The most common admission diagnoses were sepsis (21.7%), prematurity (21.3%), and respiratory distress syndrome (13.3%). The distribution of diagnoses varied by DS status, with prematurity and respiratory conditions more prominent among those with DS. Congenital anomalies were uncommon (6.3%). Antibiotic use was high overall (96.7%) and increased slightly with the number of DS. No blood cultures were performed in the sample. Most neonates survived to discharge (86%), with mortality highest among those with ≥2 DS (8%) compared to those without DS (2%) (see Table 1).

### Patterns of antibiotic use and compliance

A total of 290 patients received antibiotic treatment. The most common antibiotics prescribed upon admission were Ampicillin (93.8%) and Gentamicin (91.4%), followed by Ampicillin+cloxacillin (6.9%). Other prescribed antibiotics accounted for 2.7% together. A change in antibiotic regimen during admission occurred in 51 neonates (17.6%), while 239 (82.4%) continued their initial treatment. Among those who underwent a change, Ampicillin+cloxacillin was the most frequently used combination antibiotic (66.7%), followed by Cefotaxime (31.4%), Ampicillin (31.4%), Ceftriaxone (19.6%), Gentamicin (17.6%), Metronidazole (5.9%), and Meropenem (3.9%) (Table 2).

**Table 2:** Antibiotic prescription among all screened participants, independent of the number of DS.

Antibiotic use	Number of patients, *n* (%)
Receiving antibiotics	290 (100)
Antibiotic at admission	
Ampicillin	272 (93.8)
Gentamicin	265 (91.4)
Ampicillin + cloxacillin	20 (6.9)
Ceftriaxone	4 (1.4)
Metronidazole	2 (0.7)
Cefotaxime	1 (0.3)
Benzylpenicillin	1 (0.3)
Change in antibiotics	
Yes	51 (17.6)
No	239 (82.4)
Antibiotics changed to (51 (17.6%))	
Ampicillin	16 (31.4)
Gentamicin	9 (17.6)
Ampicillin + cloxacillin	34 (66.7)
Ceftriaxone	10 (19.6)
Metronidazole	3 (5.9)
Cefotaxime	16 (31.4)
Meropenem	2 (3.9)

**Figure 1:**
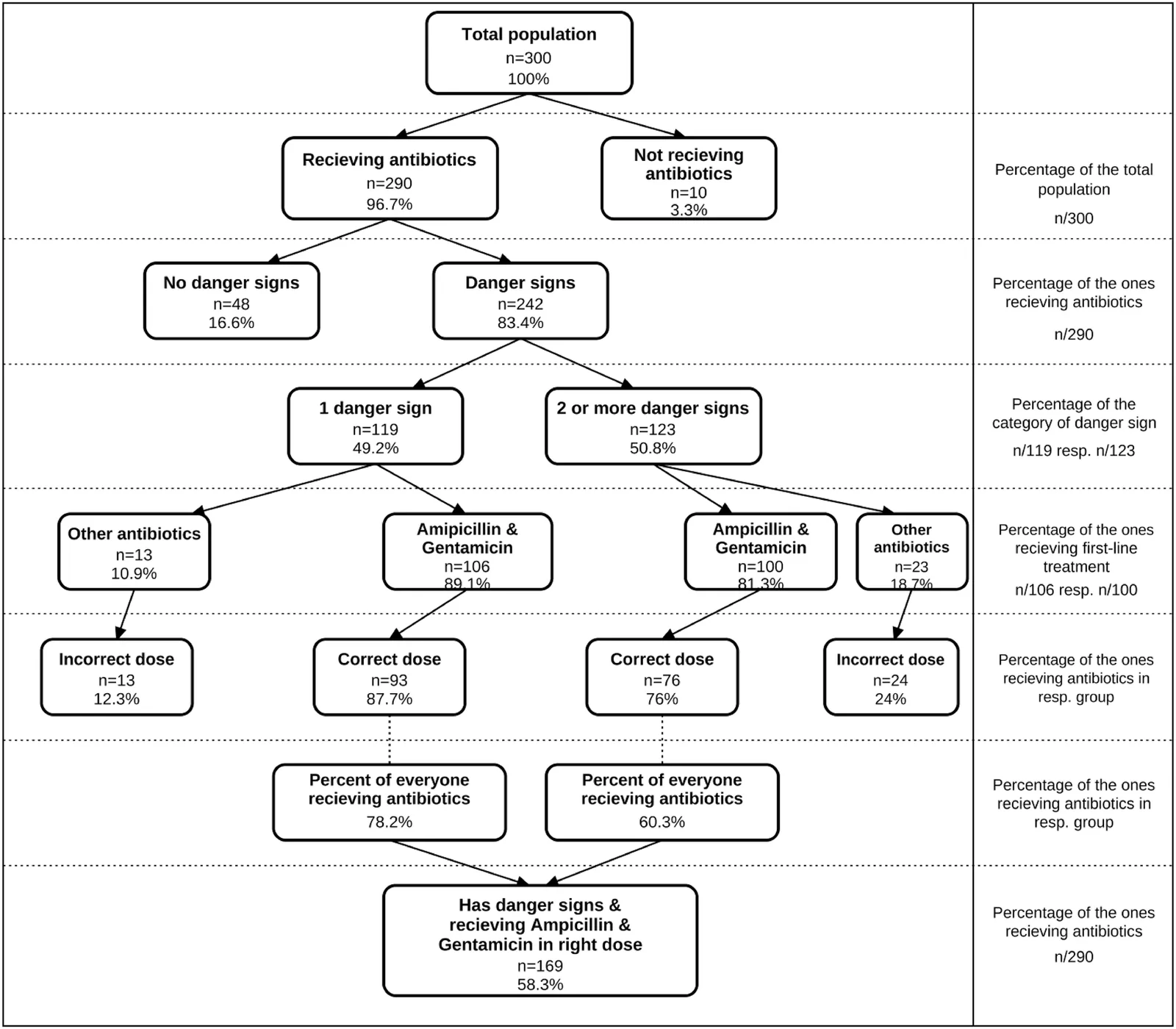
Flowchart of the population and use of antibiotics related to indication, type and dose.

Overall, 96.7% of the study population received antibiotics. Of these, 16.6% did not have any DS and therefore no clinical indication for antibiotic treatment (Figure 1). Among infants receiving antibiotics who had at least one DS (83.4%), the distribution between those with only one DS (49.2%) versus multiple DSs (50.8%) was nearly equal. Ampicillin and Gentamicin, recommended first-line treatment according to guidelines, were the most commonly prescribed antibiotics. Among infants with a single DS, 89.1% received only this combination, compared with 81.3% of those with multiple DS. Furthermore, correct dosing was achieved in 87.7% of neonates with one DS, and 76% in the group with multiple DSs. In total, out of the patients with at least one DSs, 169 (58.3%) received the correct type and dose of antibiotics.

### PSBI cases and antibiotic use

**Figure 2:**
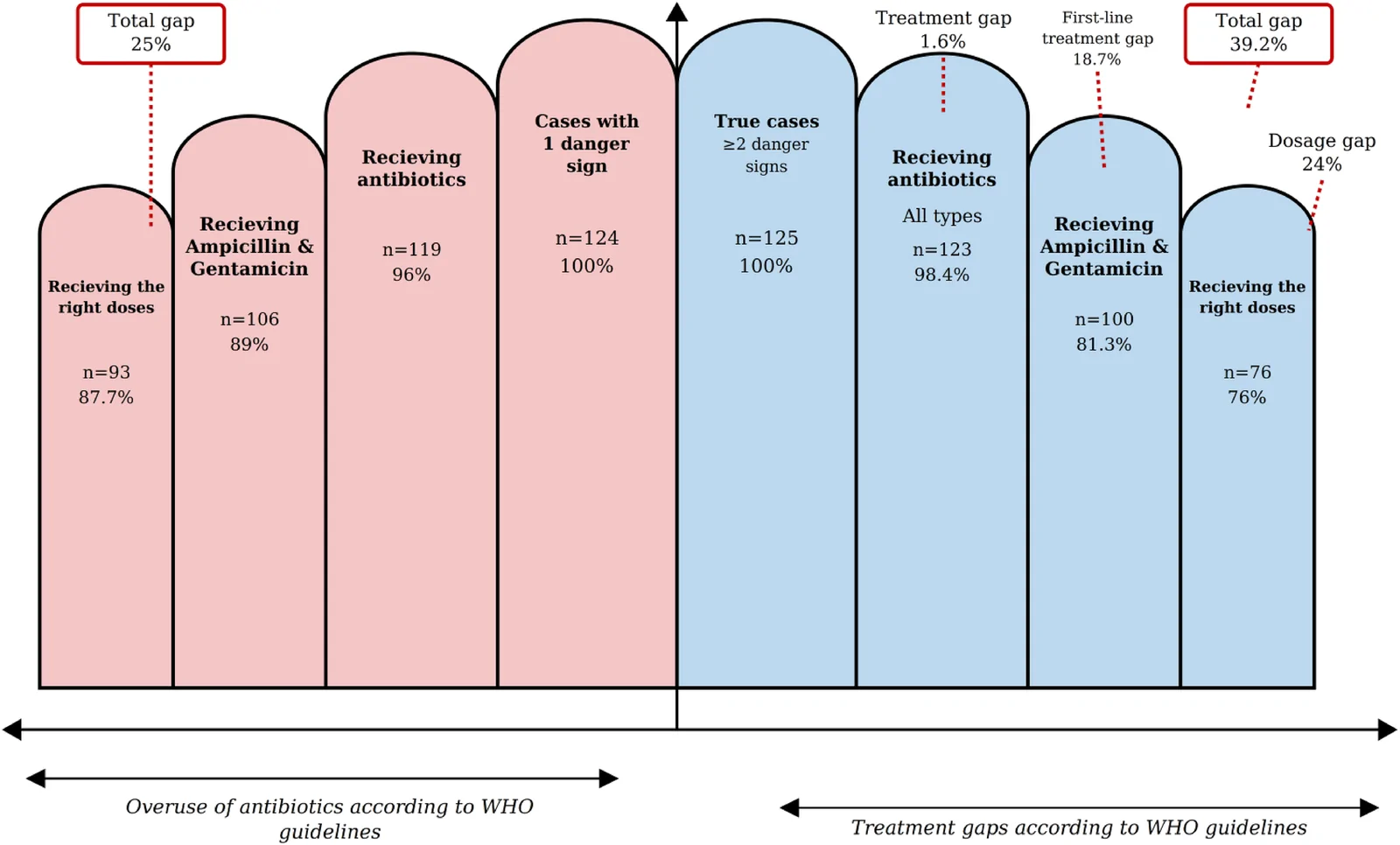
Treatment cascade for neonates with danger signs of PSBI.

Among the 125 participants who met WHO criteria for PSBI, 123 (98.4%) received some form of antibiotic treatment, leaving a treatment gap of 1.6%. First-line therapy with Ampicillin and Gentamicin was administered to 100 participants (81.3%), resulting in an 18.7% gap relative to all PSBI cases. Correct dosing of both first-line antibiotics was achieved in 76 participants (60.8%), indicating a 24% gap among those receiving the combination. Among cases presenting with only one DS (*n* = 119), 96% received antibiotics, of whom 89% (*n* = 106) were treated specifically with Ampicillin and Gentamicin. Correct dosing was achieved in 87.7% (*n* = 93) of these cases, corresponding to a total treatment gap of 25% (see Figure 2).

Overall, these findings demonstrate high rates of antibiotic coverage among PSBI cases but reveal notable gaps in adherence to recommended first-line treatment and correct dosing. Additionally, even in non-severe cases with a single DS, antibiotics were frequently prescribed, indicating potential overuse relative to WHO guidelines.

### Predictors of compliance among infants with ≥2 danger signs

Logistic regression analysis was conducted to identify factors associated with compliance in neonates presenting with ≥2 DSs, as summarised in Table 3. Age in days was significantly associated with compliance, with older infants showing slightly lower odds for compliance (OR = 0.84, 95% CI 0.72–0.98, *p* = 0.028). The number of presented DSs showed a strong and significant positive association with compliance (OR = 7.96, 95% CI 4.76–13.30, *p* < 0.001), indicating that neonates with a higher number of DS were substantially more likely to receive treatment in accordance with recommended guidelines.

**Table 3:** Potential predictive factors among neonates fulfilling WHO’s criteria of PSBI and show compliance with type and dose of antibiotics.

Factors	Odds ratio (OR)	95% CI	*p*-value
Male sex	0.68	0.33–1.37	0.28
Age (days)	0.84	0.72–0.98	0.028
Weight (gram)	1	1–1	0.18
Gestational age	1.12	0.98–1.28	0.10
Number of DS	7.96	4.76–13.30	<0.001

CI, confidence interval; DS, danger signs.

### Association between neonatal diagnoses and compliance

The logistic regression model examining the association between specific neonatal diagnoses and compliance with recommended antibiotic type and dosing among infants presenting with two or more DSs is presented in Table 4. Two diagnoses were significant predictors of compliance. Infants diagnosed with HIE had markedly increased odds of receiving treatment in accordance with guidelines (OR = 4.74; 95% CI, 1.13–19.89; *p* = 0.033) compared with those without HIE. Similarly, birth asphyxia was strongly associated with higher compliance (OR = 6.23; 95% CI, 1.30–29.83; *p* = 0.022), indicating that these clinically severe conditions were more consistently recognized and managed according to recommended protocols. Several other neonatal morbidities showed trends toward association but did not reach statistical significance. Prematurity was negatively associated with compliance (OR = 0.36; 95% CI, 0.12–1.08; *p* = 0.067), suggesting that preterm infants may be less likely to receive fully guideline-adherent care, although this did not meet conventional significance thresholds. NRDS showed a borderline positive association (OR = 2.85; 95% CI, 0.99–8.21; *p* = 0.052). Conditions such as sepsis, jaundice, macrosomia, LBW, and “other” diagnoses showed no meaningful relationships with compliance, with wide confidence intervals reflecting limited precision (Table 4).

**Table 4:** Predictor analysis among participants fulfilling WHO’s criteria of PSBI and with compliance for correct antibiotic type and dose.

Diagnosis	Odds ratio (OR)	95% CI	*p*-value
Sepsis	1.12	0.34–3.67	0.847
Prematurity	0.36	0.12–1.08	0.067
NRDS	2.85	0.99–8.21	0.052
Jaundice	0.83	0.26–2.62	0.755
Macrosomia	0.26	0.03–2.47	0.242
HIE	4.74	1.13–19.89	0.033
LBW	0.74	0.12–4.55	0.748
Birth asphyxia	6.23	1.30–29.83	0.022
Not recorded	1.03	0.28–3.73	0.969
Others	1.29	0.30–5.53	0.733

CI, confidence interval; NRDS, neonatal respiratory distress syndrome; HIE, hypoxic ischemic encephalopathy; LBW, low birthweight.

## Discussion

In this study, we evaluated adherence to recommended antibiotic therapy among neonates admitted with suspected or confirmed infection at MRRH and explored factors associated with guideline-compliant care. Overall antibiotic coverage was high, with 96.7% of neonates receiving treatment, including 16.6% without any WHO-defined DS, indicating potential overtreatment. Among neonates with at least one DS, only 58.3% received both the recommended antibiotic type and correct dosing, highlighting persistent treatment gaps. Compliance with guideline-recommended therapy was strongly influenced by clinical severity: neonates presenting with multiple DS or severe conditions such as HIE and birth asphyxia were more likely to receive appropriate care. In contrast, less severe presentations, prematurity, and single DS were associated with lower adherence or over-prescription. The absence of blood culture testing underscores a critical diagnostic limitation, restricting opportunities for targeted therapy and effective antimicrobial stewardship.

Antibiotic overuse was common, particularly among neonates with a single or no DS. Almost half of neonates with only one DS received antibiotics, a group at the threshold of treatment indication. Uganda's national guidelines recommend treatment for a single DS, whereas WHO guidelines require at least two. It is therefore important to note that antibiotic use in neonates with a single DS constitutes overuse relative to WHO criteria, but is not necessarily inappropriate according to Uganda's national guidelines; these two reference points should be distinguished when interpreting adherence findings. This discrepancy may reflect a more liberal approach in Uganda, potentially influenced by resource limitations, risk tolerance, and the desire to prevent severe outcomes. Despite high overall coverage, gaps in adherence to recommended type and dosing were observed even among confirmed PSBI cases, underscoring the need for standardized stewardship strategies.

Specific neonatal diagnoses, particularly HIE and birth asphyxia, were strongly associated with guideline-adherent treatment, suggesting that clinicians prioritize clearly severe or life-threatening conditions. In contrast, prematurity showed a trend toward lower adherence, indicating that preterm infants may receive less consistent guideline-based care, possibly due to clinical complexity or uncertainty.

### Global health implication

Several studies from Uganda and other LMICs highlight persistent gaps in adherence to guidelines on the prescription of antibiotics. A study conducted between 2016 and 2018 in eastern Uganda among outpatients of all age groups found that 82.6% of antibiotic prescriptions did not comply with national guidelines, with an even higher rate of non-compliance observed among children under 12 years of age (24). These findings are consistent with the results of the present study and underscore the widespread irrational prescribing practices of antibiotics in Uganda. Similarly, a multicenter study of inpatients across 13 facilities in Uganda found that 74% of inpatients received antibiotics, yet only 30% of prescriptions complied with guidelines. Antibiotic use was higher in public hospitals than in private facilities, reflecting systemic prescribing patterns (25).

Differences between WHO guidelines and Ugandan national guidelines may contribute to the observed discrepancies. The Ugandan guidelines take a more liberal approach to initiating antibiotic therapy, whilst the WHO recommendations are more restrictive. However, there is evidence that this strategy may encourage overtreatment. For example, a study at Mulago Hospital found that among newborns with clinical signs of sepsis (defined as at least one warning sign), only 12.8% had a culture-confirmed infection (26).

Concerns regarding AMR further complicates prescribing practices. A large-scale study of 7,000 Ugandan infants reported high resistance to the WHO's first-line antibiotics, with 40.7% of Gram-positive and 55.8% of Gram-negative isolates being resistant to both ampicillin and gentamicin (27). This high burden of resistance could partly explain the deviations from recommended treatment regimens observed in clinical practice.

The perspectives of healthcare providers also highlight systemic challenges in adhering to guidelines. A study across 32 healthcare facilities in Uganda found that whilst most clinicians recognized AMR as a major health threat, 63% reported having little confidence that their hospitals had effectively implemented antibiotic stewardship programs (ASPs). Whilst basic measures such as treatment guidelines were generally available, more advanced components—including resistance surveillance—were inadequately implemented or entirely absent (28). These findings highlight the gap between policy intentions and actual practice and underscore the need for strengthened stewardship structures, improved diagnostic capabilities and targeted support for healthcare workers to optimize antibiotic use.

Low adherence to antibiotic guidelines is not a phenomenon limited to Uganda but represents a common challenge in LMICs. Notably, poor adherence can reflect both excessive and inadequate use of antibiotics. A study in Zimbabwe and Malawi examined adherence to treatment guidelines for neonatal sepsis in over 10,000 newborns across three hospitals. Although 64%–82% of newborns met the national guideline criteria (and up to 77%–91% met the WHO criteria) for empirical antibiotic therapy, only 38%–44% in Zimbabwe and 41% in Malawi received antibiotics on admission. It is important to highlight that mortality was lower among newborns who did not receive antibiotics despite meeting the guideline criteria, suggesting possible limitations regarding the predictive accuracy and contextual relevance of current guidelines in resource-poor settings (29).

### Methodological considerations

This study adopted a census approach, in which all newborns admitted during the study period were included, to obtain a comprehensive overview of clinical assessments and minimize selection bias, thereby ensuring that the results accurately reflect the population in this setting. There was unrestricted access to patient records, and a large amount of information was available, which made it possible to complete the data collection instrument in full. Another strength of this study is its focus on MRRH, a setting that has been underrepresented in the literature to date.

However, there are some limitations to consider. As this is a retrospective study, it relies entirely on existing records and their quality and completeness. Furthermore, this study assessed compliance with national guidelines based on documented clinical DS, which depend to some extent on the subjective judgement of medical staff. The absence of microbiological confirmation limits the certainty of the actual infection diagnosis and thus compliance with the guidelines. Future studies incorporating laboratory confirmation could enable a more accurate assessment of guideline adherence. Simultaneously, these studies could also benefit from a larger sample size, which increases statistical power, improves estimation accuracy, and enhances the overall reliability and generalizability of the results. The absence of blood cultures in this cohort reflects the real-world constraints at MRRH: blood culture equipment and consumables are not routinely available in the NICU due to cost limitations, inadequate infrastructure, and the absence of a dedicated on-site microbiology service, consistent with resource constraints reported at other public referral hospitals in Uganda. Additional limitations include potential missing data in some clinical variables, the single-centre design which restricts generalisability, a short two-month study window which may not capture seasonal variation, and the lack of clinical outcome linkage, meaning it was not possible to assess whether non-adherent cases experienced worse mortality or morbidity compared to adherent cases.

The study sample for this study was drawn from one single hospital and from the same unit, that may limit the generalisability to other settings. The participants were all neonates, and caution is needed when applying these results to a broader population. Contextual factors such as local healthcare policies and cultural practices may influence the applicability of these findings to other regions or countries. The socio-demographic profile of the participants demonstrates a relative diverse neonatal population within the MRRH catchment area. The study included both sexes in near-equal proportions and spanned the full neonatal age range from 1 to 28 days. Birthweights ranged from <1,500 g to >4,000 g, with representation of low-birthweight, normal-weight, and macrosomic infants. Although the majority were born in hospital, a small proportion were delivered at home or in clinics, reflecting variation in access to perinatal care.

Although relatively high adherence to national guidelines was observed based on documented danger signs, the absence of microbiological confirmation means that some neonates may not have had true infections. Therefore, guideline adherence should be interpreted with caution, as documented compliance may not fully reflect appropriate antibiotic use.

## Conclusion

This study demonstrates a high overall rate of antibiotic use among newborns at the MRRH, with evidence of both overuse in less severe cases and gaps in adherence to recommended treatment regimens in severe cases of infection. Clinical severity, particularly multiple DS and diagnoses such as HIE or birth asphyxia, was a strong predictor of adherence to treatment, whilst prematurity and milder clinical presentations were associated with lower adherence or overtreatment. These findings underscore the need for enhanced antibiotic stewardship, improved diagnostic capabilities and targeted support for clinicians to optimize antibiotic use in newborns, ultimately reducing the risk of AMR and improving neonatal outcomes in resource-limited settings. Specifically, introduction of quality improvement cycle on antibiotic dosing, consideration of once-daily gentamicin dosing protocols to simplify administration, and investment in blood culture capacity at MRRH are urgently needed to improve guideline adherence for small and sick newborns.

## Data Availability

The raw data supporting the conclusions of this article will be made available by the authors, without undue reservation.
